# Dual‐System Collaborative Model of Prosocial Risky Behavior and Cognitive Computation: A Review

**DOI:** 10.1002/pchj.822

**Published:** 2024-12-26

**Authors:** Changlin Liu, Boqiang Zhao, Youlong Zhan, Ping Hu, Xiaoqin Mai

**Affiliations:** ^1^ Department of Psychology Renmin University of China Beijing China; ^2^ Department of Psychology Hunan University of Science and Technology Xiangtan China

**Keywords:** cognitive computation, cross‐culture, dual‐system collaborative model, prosocial risky behavior

## Abstract

Prosocial risky behavior (PRB) refers to actions taken at personal risk for the benefit of others or societal welfare, combining risk‐taking with prosocial intent, and involving the integrated processing of individual risk and social preferences. Building upon the review and evaluation of the definitions of PRB, existing research tools, theoretical models, and neural mechanisms, this paper elucidates the synergistic interaction and mechanisms of the emotional drive and cognitive reasoning systems in PRB. It constructs a dual‐system collaborative model for PRB. Furthermore, to address the shortcomings of existing PRB research tools, such as limited cross‐domain applicability and low reliability, this paper designs a PRB research paradigm within the economic decision‐making domain. Combined with the dual‐system collaborative model of PRB, this paper proposes a cognitive computational modeling concept for PRB and preliminarily verifies its reliability. Future research should conduct cross‐cultural studies, utilizing cognitive neuroscientific technologies, to explore the cultural differences in the mechanisms underlying PRB, thereby enhancing the cross‐cultural interpretive power of the constructed dual‐system collaborative model of PRB. This broadens the theoretical explanatory pathways and research dimensions of PRB.

## 1 Introduction

In real life, prosocial behaviors often come with risks, and when individual health and safety are at stake, the motivation to protect oneself may conflict with the motivation to protect others (Bixter and Luhmann 2014; Vieira et al. 2020). For example, during the “12·18” earthquake disaster in Gansu, China, a series of “in‐danger assistance” behaviors emerged, such as People's Liberation Army soldiers risking their lives to rush to the frontlines for rescue. Prosocial risky behavior (PRB) refers to actions undertaken at personal risk for the benefit of others or societal welfare (Do, Moreira, and Telzer 2017). Typically, the motivation for such behavior is the benefit of others or the collective rather than the individual, and participants often face potentially unknown costs, such as health risks or financial losses. Consequently, it is usually regarded as a special form of prosocial behavior that involves risk (Zhan et al. 2023). Exploring the mechanisms underlying PRB can help to curb and regulate negative behavioral tendencies in humans, further promoting a positive transformation, and thus has garnered widespread attention from researchers (Arfer, Bixter, and Luhmann 2015; Bereczkei, Birkas, and Kerekes 2010; Minwoo, Sunhae, and Hackjin 2018; Volz et al. 2017).

In recent years, the academic community has conducted extensive research on PRB. Developmental psychologists were among the first to integrate cognitive neuroscience techniques to examine and compare the cognitive and neural mechanisms of singular negative risky behaviors and positive prosocial behaviors. They conceptualized PRB to reveal the dual positive dimensions of adventurousness and prosociality exhibited by individuals in specific actions (Do, Moreira, and Telzer 2017). Compared to singular prosocial behaviors, the distinctive feature of this behavior lies in the uncertainty of its potential costs; whereas, in contrast to singular risky behaviors, its uniqueness lies in the potential to yield positive benefits for others or society through its execution (Zhan et al. 2023). Subsequently, researchers developed and revised the Chinese version of the “Adolescent Prosocial Risky Behavior Scale,” thereby creating conditions for studying PRB through longitudinal tracking (Skaar, Christ, and Jacobucci 2014; Dou et al. 2020). Researchers utilized this scale to investigate the mechanisms underlying adolescent PRB further, influenced by factors such as parenting styles, subjective social status, and peer relationships within social contexts or background information (Feng, Dou, and Tang 2024; Zhang et al. 2024). The questionnaire method possesses a certain degree of structure and theoretical underpinning, which can effectively examine the various sub‐components of PRB. However, due to its inherent high subjectivity, it is challenging to assess individual actual PRB tendencies accurately. In addition to questionnaire methods, research paradigms such as the fire escape task and prosocial risky dilemma priming task have provided a novel approach within the social decision‐making field to examine PRB under laboratory conditions (Gross et al. 2020; Gu, Liu, and Cui 2019; Zanon et al. 2014). Researchers, based on these paradigms, manipulate variables such as risk levels, situational urgency, peer presence, and the social distance of decision targets to explore further whether PRB is influenced by decision‐making contexts (Liu et al. 2023; Zhan et al. 2022). The advantage of PRB research paradigms in social decision‐making is the wide array of materials that can be utilized. By varying the narrative contexts, decision‐making scenarios, outcomes, and other elements, participants can project their internal beliefs in their responses, reflecting individual behavioral tendencies with significant flexibility and selectivity. However, the complexity of the design content also increases the number of irrelevant variables that need to be controlled. These experimental paradigms are often based on hypothetical situations, which may lack the direct experience and emotional investment of individuals, potentially affecting their decision‐making responses. Moreover, human decision‐making behavior exhibits domain specificity. In addition to the social decision‐making perspective, it is necessary to develop experimental paradigms with more refined content and real monetary incentives from an economic decision‐making perspective. This approach will allow for a comprehensive exploration and comparison of the genuine response tendencies and mechanisms of PRB from different domain perspectives. Furthermore, the refined PRB research paradigms within the economic decision‐making domain can be effectively matched with cognitive computing modeling technology, which aids in quantifying and explaining the cognitive mechanisms of PRB. However, there is currently a lack of a scientifically rigorous cognitive computational framework for PRB, and no studies have yet effectively integrated the two.

Regarding the cognitive processes of PRB, there are currently three main explanatory theoretical models: the rational reasoning model, the social intuition model, and the dual‐process model. The rational reasoning model posits that PRB is a conscious analytical reasoning process, where the cognitive system represents and processes prosocial risky dilemma information while regulating the resulting emotional responses (Paxton and Greene 2010). The social intuition model emphasizes that the role of emotion in PRB far outweighs that of cognition, with conscious reasoning processes occurring only after PRB has been made, and serving merely to provide supplementary explanations (Huebner, Dwyer, and Hauser 2009). The dual‐process Model suggests that there are two systems in human prosocial risk cognition, some prosocial risky dilemmas are processed with a greater cognitive component, while others involve more emotional factors. The former is a controlled, effortful cognitive process, and the latter is a parallel, automated processing mechanism (Zhan et al. 2023). However, existing theoretical models have limitations in their explanatory power under specific conditions and tend to overlook the dynamic interaction between cognition and emotion in PRB.

Moreover, as research progresses, scholars utilize electrophysiological and brain imaging techniques to investigate and elucidate the neural mechanisms underlying PRB from both temporal characteristics and neural foundations (Do, Moreira, and Telzer 2017; Zhan et al. 2023). On the one hand, specific electroencephalographic components such as P2, P3, and late positive potential (LPP) demonstrate the dynamic interplay between emotion and cognition in PRB, from emotional response to cognitive reasoning, revealing the brain's specific temporal processing characteristics. On the other hand, neural networks and subsystems involved in social emotion, social reward, and cognitive control, including but not limited to key brain regions like the amygdala, ventral striatum (VS), and ventromedial prefrontal cortex (vmPFC), provide important perspectives for understanding the neural mechanisms of PRB through their individual activation levels and functional connectivity (Zhan et al. 2023). However, current research methodologies have limitations, often focusing on individual electroencephalographic components or isolated activation of specific brain regions. A comprehensive understanding of the connection between cognitive processes and brain functional networks underlying PRB is yet to be formed. There is a deficiency of a model that systematically elucidates the synergistic mechanisms between the cognitive reasoning system and the emotional drive system in PRB, thereby providing a more holistic and comprehensive explanation for the occurrence and development of PRB.

Therefore, based on previous research, this paper first reviews the definitions of PRB, existing research tools, and theoretical models, providing a theoretical explanation for such behaviors. Second, integrating relevant neural mechanisms, this paper constructs a dual‐system collaborative model of emotional drive and cognitive reasoning, aiming to offer a novel and comprehensive dynamic perspective on the mechanisms of PRB. Additionally, this paper designs a research paradigm for PRB within the economic decision‐making domain, specifically a risk‐assisting task with real monetary rewards, proposes a corresponding cognitive computational modeling concept for PRB, and preliminarily validates the effectiveness of the computational model. Finally, this paper outlines future research directions, starting from a cross‐cultural comparative perspective, investigating the cultural differences in PRB and their underlying mechanisms from three aspects: cultural values, self‐concept, and cognition orientation, aiming to enhance the cross‐cultural applicability of the dual‐system collaborative model of PRB and to further reveal the deeper nature of PRB. This broadens the theoretical explanations for the mechanisms underlying PRB, expands the methodological dimensions for the study of PRB, and provides methodological and instrumental support for further revealing the cognitive mechanisms of PRB. On a practical level, it lays a theoretical foundation for the intervention of PRB.

## 2 Theoretical Models of Prosocial Risky Behavior

As a complex form of prosocial behavior, a deep exploration of the underlying cognitive mechanisms of PRB is crucial for revealing its patterns of occurrence and development. Existing research has demonstrated that variables such as emotional responses, cognitive control abilities, and specific contexts can significantly influence PRB (Liu et al. 2023; Zhang et al. 2024; Zhan et al. 2022). Despite this, the theoretical understanding of PRB remains limited, with many studies still relying on theoretical models related to social value decision‐making to explain the phenomena. Therefore, this paper first summarizes previous theoretical models of PRB to form a theoretical understanding at the conceptual level.

### 2.1 Rational Reasoning Model

The rational reasoning model posits that cognitive development is a necessary condition for the evolution of individual PRB reaction modes primarily made through reasoning and rational contemplation. The occurrence of PRB is a conscious analytical reasoning process, with emotional responses arising after rational analysis. The cognitive system represents and processes information regarding prosocial risk dilemmas while regulating the resulting emotional reactions (Paxton and Greene 2010). When engaging in PRB, individuals first gather and organize information within prosocial risky dilemmas, then make decisions after fully representing and processing this information (Zhong et al. 2018). According to this theoretical model, PRB involves four stages: First, support for behaviors or social norms related to PRB; second, considering such PRB norms as general standards suitable for all rational agents; third, evaluating the feasibility of a society based on these PRB norms; and finally, if feasible, whether one is willing to incorporate these PRB norms into their behavioral standards (Bucciarelli et al. 2008). Additionally, from the perspective of the rational reasoning model, individuals exhibit two types of processing tendencies when dealing with prosocial risky dilemmas: automatic social cognition and controlled social cognition. Automatic social cognition refers to individuals relying on heuristics and simple rules to make judgments during the PRB process, which is relatively fast and effortless. In contrast, individuals with a controlled social cognition orientation tend to consider multiple options and situational characteristics, with PRB being the result of deliberate contemplation (Ajzen 1991; Zhong et al. 2018). The rational reasoning model explains the occurrence of PRB from the perspective of cognitive processing, suggesting that individuals engage in PRB based on rational reasoning. However, this model also has its shortcomings. It posits that there is no emotional involvement during the process of PRB and that emotions arise only after PRB is completed, overly emphasizing the importance of cognition and neglecting the significant impact of emotions on PRB. Previous research has found that urgent prosocial risk dilemmas can elicit strong feelings of guilt aversion in individuals, who may then quickly engage in PRB to alleviate this negative emotion (Minwoo, Sunhae, and Hackjin 2018). Thus, relying solely on the rational reasoning model to explain the mechanisms underlying PRB under certain special conditions has certain limitations.

### 2.2 Social Intuition Model

The social intuition model suggests that emotions serve as heuristic tools for simplified judgment, guiding individuals in engaging in PRB as a significant influencing factor (Bright and Goodman‐Delahunty 2006; Zheng, Chen, and Mai 2024). Negative emotional experiences evoked by prosocial risk dilemmas are an important motivational source for PRB (Volz et al. 2017). When faced with prosocial risky dilemmas, individuals undergo a range of negative emotions, including loss aversion induced by risk information in the dilemma (Zhan et al. 2022) and guilt aversion induced by the appeal for help (Rand, Epstein, and Thomas 2014). At this juncture, the individual initial intention may be to alleviate their negative emotions by engaging in risk‐ignoring PRB or by choosing not to help, with the subsequent acquisition of a good social reputation being merely an additional benefit (Zhan et al. 2023; Zheng, Chen, and Mai 2024). Furthermore, the model emphasizes that the role of emotions in PRB far exceeds that of cognition, with conscious reasoning processes occurring only after the completion of PRB, serving merely to provide supplementary explanations (Haidt 2001). When engaging in PRB, individuals are typically first driven by emotional intuition to make choices, with rational reasoning occurring afterward, primarily to find suitable justifications for PRB, to persuade others, and to reflect on and evaluate the outcomes of their choices (Haidt 2001). However, the social intuition model may overemphasize the role of intuition in PRB without giving due consideration to deliberate reasoning. This perspective might overlook individual capacity for in‐depth thinking and evaluation when confronted with prosocial risk dilemmas. Moreover, the model disregards the complexity of PRB, the judgment and reasoning processes involved in PRB may be more intricate than described by social intuition theory. For instance, PRB may involve multiple aspects such as setting personal goals, determining responsibilities, weighing action choices, and assessing the quality of PRB (DeTienne et al. 2021). Therefore, while this model offers valuable insights into the process of PRB, it has significant explanatory shortcomings regarding the cognitive mechanisms of PRB.

### 2.3 Dual‐Process Model

The dual‐process model of PRB suggest that its occurrence involves two independent processing systems (Zhan et al. 2023). On the one hand, from the perspective of evolutionary psychology, individuals may engage in PRB due to self‐centered prosocial motivation (Fehr and Fischbacher 2003; Paramita, Septianto, and Tjiptono 2020). For example, individuals may make altruistic choices to establish a good reputation (Bereczkei, Birkas, and Kerekes 2010). Although such behavior may entail potential losses for the individual, under certain conditions, the spread of reputation can bring more long‐term benefits, especially when observed or evaluated by others (Liu et al. 2023). The individual assessment and valuation of specific benefits may trigger the occurrence of such behavior, reflecting the outcome of rational analysis and cognitive reasoning processes at the conscious level in humans (Bixter and Luhmann 2014; Zhan et al. 2023). When discussing how individuals balance unknown risks with potential benefits in prosocial risky dilemmas, the social reference point theory offers an explanatory framework. This theory posits that an individual PRB is driven not only by the goals they pursue but also moderated by their baseline standards (Van Knippenberg, Van Dick, and Tavares 2010; Zhan et al. 2023). The goal represents the benefits that an individual expects to gain from PRB, while the baseline defines the minimum expected value of those benefits (Locke and Latham 2002). Individuals aim to maximize the probability of achieving their goals and minimize the risk of failing to meet their baseline expectations when making prosocial risk decisions, thereby pursuing the greatest benefits while ensuring a minimum level of gain. Furthermore, risk sensitivity theory further explains how individuals set goals and baselines based on their intrinsic needs and posits that these needs facilitate the occurrence of PRB (Song, Xu, and Xing 2017). Individual pursuit of specific needs to be satisfied serves both as the goal of their actions and as their baseline. When there is a significant discrepancy between an individual actual situation and their expected goals or baseline, such as a damaged reputation or low social status, this gap may enhance their prosocial inclination and willingness to take risks, thereby prompting them to adopt a decision‐making strategy that appears altruistic but is essentially motivated by self‐interest considerations (Xie and Lu 2014).

On the other hand, according to the social intuitionist theory, human PRB is a parallel and automated psychological process, in which rational analysis typically occurs after decision‐making, serving to complement and interpret intuitive judgments (Crockett et al. 2014; Minwoo, Sunhae, and Hackjin 2018). People may continue to exhibit PRB because there is an inherent motivation to sacrifice personal interests for the welfare of others (Zhan et al. 2023). When making rapid intuitive judgments about prosocial risky dilemmas, existing research has confirmed that under the influence of situational factors, individuals exhibit either a prosocial motivation to benefit others without fear of risk or a complete aversion to loss indicative of risk avoidance (Rand, Epstein, and Thomas 2014; Vugt and Iredale 2013). For example, in emergencies, intense feelings of guilt aversion can be triggered. To mitigate this negative emotion, psychological defense mechanisms may drive individuals to engage in more PRB (Minwoo, Sunhae, and Hackjin 2018; Volz et al. 2017). Furthermore, the closeness of interpersonal relationships, such as social distance, can also modulate the way situational factors influence PRB. When faced with high‐risk helping situations, individuals often exhibit more extreme altruistic decision‐making tendencies towards their relatives or close friends (Zhan et al. 2022). Similar studies have found that, compared to strangers, individuals are significantly more likely to engage in PRB towards their relatives under high‐risk conditions, specifically showing a 52% higher probability. Additionally, significantly stronger functional connectivity is detected in the frontal and temporoparietal junction (TPJ) areas of the brain. The activation and connectivity strength of these key brain regions are significantly positively correlated with PRB (Cui et al. 2022). This indicates that when the relationship between the person in need and the individual is closer, the individual perception of others' negative emotions intensifies, while their perception of potential loss risks diminishes. Consequently, the intensity of loss aversion emotions triggered by risk information weakens, leading the individual to potentially engage in PRB quickly. Thus, the occurrence of such behavior is a product of the situation‐induced strong feelings of guilt aversion or weak feelings of loss aversion.

The aforementioned theoretical models elucidate the mechanisms underlying PRB from various perspectives. However, existing theoretical models have certain limitations in explaining PRB. Influenced by situational characteristics, human social decision‐making exhibits dynamic variability. For example, in prosocial risky dilemmas with high levels of risk, individuals experience intense loss aversion emotions triggered by risk information, leading to indifferent and unhelpful choices. This risk‐avoidance tendency diminishes as the level of risk decreases (Liu et al. 2023). Furthermore, under conditions of high‐risk levels, participants experience stronger guilt aversion emotions in risk‐assisting scenarios involving acquaintances (vs. strangers). At this time, participants' decision‐making time is longer, and there is detection of larger amplitudes of the P2 electroencephalographic component associated with negative emotions and the P3 and LPP components related to cognitive reasoning (Chen et al. 2009; Zhan et al. 2022), suggesting that the processing of prosocial risky dilemma information may involve the interaction between emotional processing and cognitive reasoning. Therefore, under certain conditions, PRB cannot be reasonably explained by existing theoretical models, as cognition and emotion may not operate independently. Clarifying the synergistic interaction and mechanisms between cognitive reasoning and emotional drive in PRB may broaden the interpretive pathways of previous research on the mechanisms underlying this behavior.

## 3 Dual‐System Collaborative Model of Prosocial Risky Behavior

**FIGURE 1:**
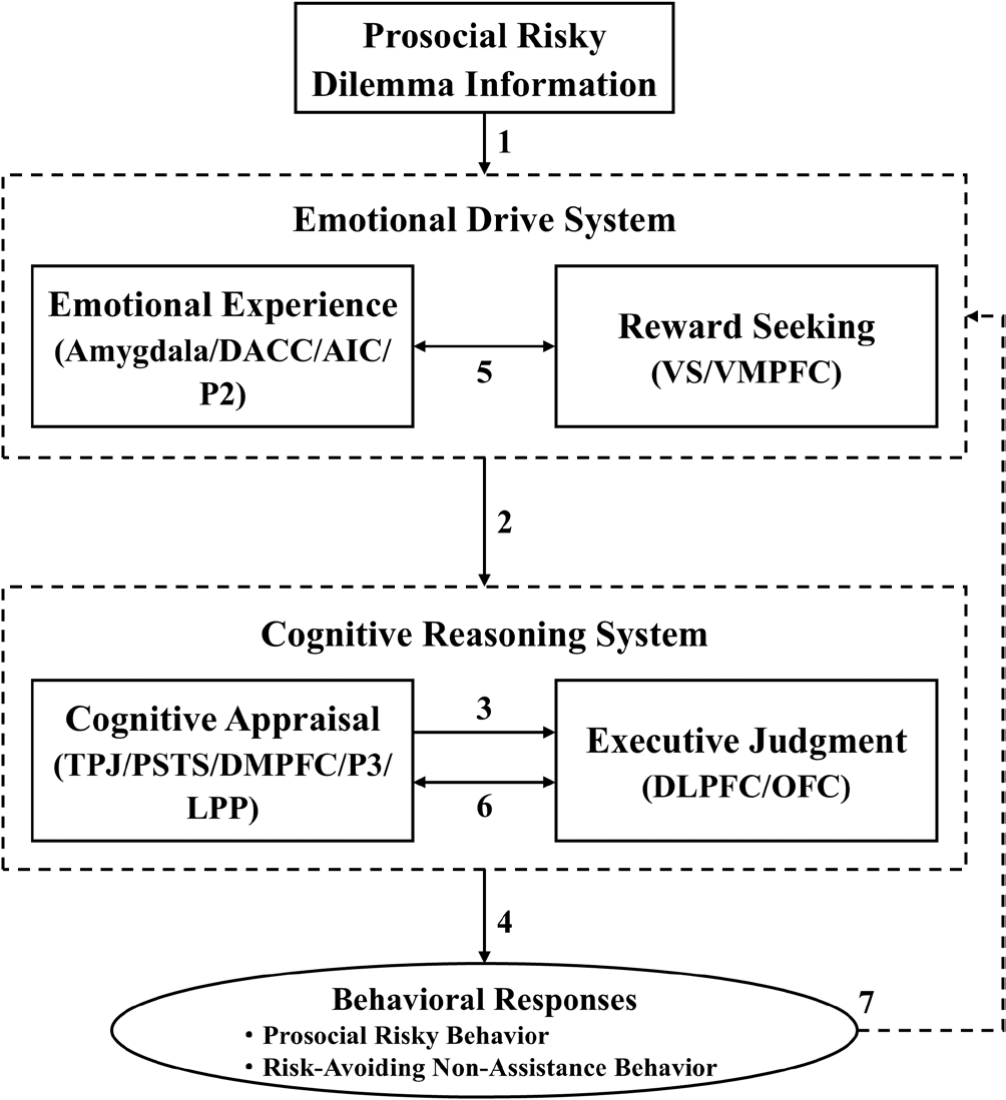
Dual‐system collaborative model of prosocial risky behavior.

Previous research indicates that the synergistic interaction of emotional drive and cognitive reasoning in human social decision‐making reflects the individual recognition of emotions and reward‐seeking information induced by the decision context, and subsequent cognitive inference of these cues (Dolcos, Iordan, and Dolcos 2011; Zheng, Chen, and Mai 2024). Prosocial risky dilemmas often elicit varying degrees of loss aversion and guilt aversion emotions in individuals (Minwoo, Sunhae, and Hackjin 2018; Zhan, Xiao, Tan, Li, et al. 2020; Zhan, Xiao, Tan, and Zhong 2020). However, there is currently a lack of a more comprehensive model to explain how individuals manage the two types of aversion emotions in prosocial risky dilemmas and the collaborative mechanism of the emotional drive and cognitive reasoning systems. Therefore, it is necessary to integrate evidence from psychological and neural levels to form a more systematic understanding. To explain the mechanisms underlying PRB from a more comprehensive perspective, based on previous PRB theoretical models and in conjunction with the neural basis of PRB, this paper clarifies the synergistic effects and mechanisms of emotional drive and cognitive reasoning in prosocial risky dilemmas and further constructs a dual‐system collaborative model of PRB (as shown in Figure 1), in an attempt to further reveal the mechanisms underlying this specific behavior.

### 3.1 Emotional Experience and Reward‐Seeking Within the Emotional Drive System

Emotion theories posit that the aversion emotions elicited by dilemma information serve as a significant driving force behind PRB (Crockett et al. 2014). Given its inherent risk and prosocial nature, prosocial risky dilemmas elicit two types of aversion emotions, loss aversion and guilt aversion (Chen, Julien, and Dreher 2022; Moreira et al. 2021). Loss aversion is triggered by risk information and reflects the emotional representation of unknown loss in prosocial risky dilemmas for individuals (Song, Xu, and Xing 2017; Zhang, Yu, and Mai 2020). Research has found that the activation level of the amygdala peaks when individuals process unknown loss risk information. This brain region's activation is particularly sensitive to loss aversion related to risk preference and is associated with the rapid encoding of risk information in dilemmas (Blakemore and Mills 2014; Hare et al. 2008). Guilt aversion is an internal experience where individuals focus on the welfare of others or society and is closely related to the level of social preference (Tan, Wang, and Cui 2017). The higher the level, the more it leads to a motivation to sacrifice personal interests for the benefit of others (Hursh and Roma 2013; Yudkin et al. 2022). For example, in urgent prosocial risky dilemmas, individuals experience intense feelings of guilt aversion (Sevinc and Spreng 2014). In urgent prosocial risk dilemmas, the dorsal anterior cingulate cortex (dACC) and anterior insula cortex (AIC) become highly active, and a greater magnitude of the P2 component is detected in the parietal and frontal regions (Telzer 2016). The activation levels of these brain areas related to the social affective neural network of PRB, as well as the intensity of electroencephalographic components, are significantly positively correlated with the levels of guilt aversion emotions induced by dilemmas (Telzer 2016).

The theoretical models of PRB suggest that individuals may engage in a series of altruistic behaviors that are ultimately self‐serving to gain some potential benefits, reflecting a self‐serving prosocial motivation (Paramita, Septianto, and Tjiptono 2020; Zhan et al. 2023). For example, public prosocial risky dilemmas can elicit strong loss aversion emotions in individuals. To avoid damaging their good reputation, individuals are inclined to take more risky actions to help others. Additionally, a larger amplitude of the P2 component is detected in the frontal lobe of their brains (Zhan, Xiao, Tan, Li, et al. 2020; Zhan, Xiao, Tan, and Zhong 2020). The magnitude of this electroencephalographic component is significantly positively correlated with the level of loss aversion emotions reported subjectively by the individuals (Zhan, Xiao, Tan, Li, et al. 2020; Zhan, Xiao, Tan, and Zhong 2020). Moreover, related neuroimaging studies have found that when individuals engage in PRB under public conditions, brain regions associated with the social reward network, such as the VS and VMPFC, exhibit stronger activation levels (Telzer 2016). The VS and vmPFC are brain regions sensitive to reward prediction and encoding. Individual anticipation of potential gains (such as reputation and cooperation) and the psychological value derived from obtaining expected benefits (such as satisfaction) may serve as reward feedback signals to further promote the occurrence of subsequent PRB (Bault et al. 2011). Therefore, emotional experience and reward‐seeking may together form the emotional drive system of PRB, and the two are closely interconnected (indicated by arrow 5). Under public (vs. anonymous) conditions, the aversion to reputation loss triggered by prosocial risky dilemmas may foster the emergence of reward‐seeking motives. Driven by psychological defense mechanisms, individuals are more likely to focus on and seek potential gain information to alleviate internal conflicts caused by loss aversion. For example, in the presence of peers, individuals are more inclined to engage in PRB, experiencing heightened aversion to reputation loss and exhibiting a more pronounced P2 component, which is significantly positively correlated with the level of loss aversion emotions, when confronted with prosocial risky dilemmas (Chen et al. 2009; Skaar, Christ, and Jacobucci 2014). After receiving peer praise, the level of individual loss aversion significantly decreases (Liu et al. 2023; Yu et al. 2022). Motivational theories explain this phenomenon, suggesting that identity motivation prompts individuals to be highly sensitive to social feedback, such as others' evaluations or their status (Zhan et al. 2023). They focus more on seeking potential benefits (such as reputation and status), and as a result, the experience of loss aversion emotions triggered by dilemma information after obtaining expected gains tends to be diminished (Bereczkei, Birkas, and Kerekes 2015; Zhan et al. 2018). Moreover, the benefits derived from engaging in PRB can further induce positive emotional experiences in individuals (Zhan et al. 2023). For example, individuals engaging in PRB report higher levels of positive emotional experiences after receiving monetary rewards or praise from others (Bereczkei, Birkas, and Kerekes 2010). Thus, the interplay between emotional experience and reward‐seeking provides an internal drive for individuals to engage in PRB.

### 3.2 Cognitive Appraisal and Executive Judgment Within the Cognitive Reasoning System

The Cognitive reasoning system encompasses two sub‐components: cognitive appraisal and executive judgment. When engaging in PRB, individuals must evaluate the two types of aversion emotions induced by prosocial risk dilemma information and weigh and calculate reward information (Zhan et al. 2023). The activity of the theory of mind sub‐neural network within the PRB social affective network system reflects an individual ability to infer the needs of others in prosocial risky dilemmas, as well as the trade‐off between potential and unknown gains. The activation of related brain regions such as the TPJ, posterior superior temporal sulcus (pSTS), and dorsomedial midline prefrontal cortices (dmPFC) reflects the interaction between emotional experience and value assessment calculations in PRB (Zhan et al. 2023). Furthermore, the amplitude of the parietal P3/LPP component reflects the attentional resources and cognitive effort individuals invest to resolve prosocial risky dilemmas (Zhan, Xiao, Tan, Li, et al. 2020; Zhan, Xiao, Tan, and Zhong 2020). However, the activation levels of the dorsolateral prefrontal cortex (dlPFC) and orbitofrontal cortex (OFC) within the cognitive control neural network are closely related to the level of cognitive executive control in individual PRB (Zhan et al. 2023). For example, scholars have indicated that individual behavioral responses in prosocial risky dilemmas rely on brain regions such as the dlPFC and OFC. The degree of activation and functional connectivity of these areas reflect the level of self‐control in individuals facing prosocial risky dilemmas (Blakemore and Mills 2014). Thus, the cognitive appraisal and executive judgment processes within the cognitive reasoning system of PRB may correspond to the theory of mind sub‐neural network, P3 component, LPP component, and cognitive control neural network, respectively participating in the evaluation and execution phases.

In addition, when facing prosocial risky dilemmas, the activation of the theory of mind sub‐neural network facilitates the activity of brain regions within the cognitive control neural network. That is, individuals heightened attention to their own emotional experiences related to PRB (both types of aversion emotions and positive emotions caused by rewards) and the potential decision values in the dilemma (gains vs. losses) can regulate their level of self‐control, thereby prompting a refined processing of executive judgment concerning the execution of PRB (Qu et al. 2015). Thus, the theory of mind and cognitive control neural networks may have a progressive relationship rather than a disjointed one in PRB. However, related research has also found that the dlPFC exerts an inhibitory effect on the TPJ during social interaction and decision‐making processes, suggesting a possible antagonistic relationship between the two (indicated by arrow 6; Zheng, Chen, and Mai 2024). For example, some studies have found that the functional connectivity between the two networks at rest is significantly negatively correlated with an individual level of social decision‐making. Moreover, when making decisions, the dlPFC transitions from a deactivated to an activated state, while the TPJ remains consistently activated (Zinchenko, Nikulin, and Klucharev 2021). Furthermore, the theory of mind sub‐neural network and cognitive control neural network related to social decision‐making may functionally exhibit a complementarity between intuition and rationality (Chen, Xu, and Wang 2020). Specifically, the former represents the rapid processing of intuition, reflecting cognitive appraisals dominated by emotions and values, while the latter represents the process of elaborate reasoning, with its role in the execution judgment of PRB manifested in the cognitive trade‐off of emotions and values.

### 3.3 Processing Pathway of the Dual‐System Collaborative Model for Prosocial Risky Behavior

Regarding the processing pathways of the dual‐system collaborative model for PRB, based on theoretical models of PRB (Bright and Goodman‐Delahunty 2006; Paxton and Greene 2010; Zhan et al. 2023), the model may involve three pathways: intuitive processing (A), rational processing (B), and feedback reinforcement (C). On the one hand, pathways A and B begin with the emotional experience sub‐component of the emotional drive system and culminate in the output of different behavioral responses in prosocial risky dilemmas through the executive judgment sub‐component of the cognitive reasoning system. Specifically, in pathway A, after individuals receive prosocial risky dilemma information (indicated by arrow 1), the two types of strong aversion emotions evoked by risk and help‐seeking information are integrated and transmitted directly from the emotional experience sub‐component to the cognitive reasoning system (indicated by arrow 2), bypassing the reward‐seeking sub‐component of the emotional drive system, and quickly output behavioral responses through the executive judgment subsystem (PRB/risk‐avoidant non‐helping behavior, indicated by arrow 4). For example, actively escaping the defensive state caused by intense internal conflict due to guilt aversion may prompt people to disregard the risks of personal interest loss, thereby providing more help to others in urgent situations with shorter response times. In this case, under the influence of psychological defense mechanisms, PRB that disregards losses may alleviate the internal conflict caused by guilt aversion emotions in individuals (Volz et al. 2017). Furthermore, prosocial risky dilemmas at high‐risk levels can induce intense loss aversion emotions in individuals. At such times, individuals are more inclined to engage in risk‐avoidant behaviors characterized by indifference and lack of assistance, thereby regulating their internal conflict state to a stable condition (Zhan et al. 2022). Pathway B involves the process of cognitive evaluation and reasoning when individuals engage in PRB. For example, research has found that when facing prosocial risky dilemmas, individuals not only experience strong emotions of loss aversion or guilt aversion (Volz et al. 2017; Zhan, Xiao, Tan, Li, et al. 2020; Zhan, Xiao, Tan, and Zhong 2020), but also exhibit larger amplitudes of the P3 and LPP electroencephalographic components in the parietal region of their brains (Sarlo et al. 2012). Emotional experience and anticipated reward information first enter the cognitive appraisal sub‐component of the cognitive reasoning system, thereby assessing the intensity of the positive emotions evoked by the two types of aversion emotions and reward information, as well as the cost–benefit information. Subsequently, the executive judgment sub‐component carries out an emotional and value trade‐off calculation (indicated by arrow 3), ultimately deciding whether to engage in PRB (indicated by arrow 4). This corresponds to the three stages of emotional experience and cost–benefit recognition, evaluation and calculation, and PRB response.

On the other hand, during the social decision‐making process, individuals continuously receive and evaluate information from both internal and external sources, thereby timely adjusting their behavioral strategies (Zheng, Chen, and Mai 2024). Concerning this model, once PRB occurs (indicated by arrow 4), the aversion emotions induced by prosocial risky dilemmas are alleviated, and the benefits obtained after helping (such as a good reputation, satisfaction, and long‐term cooperation) are fed back as reward information to the reward‐seeking sub‐component of the emotional drive system (indicated by arrow 7), thereby providing impetus for the occurrence of the next PRB. For example, research has found that praise from others and the establishment of a good reputation further promote the occurrence of PRB, and individuals subjectively report higher levels of positive emotions (Bereczkei, Birkas, and Kerekes 2010). Reinforcement learning theory explains this phenomenon: individuals continuously learn and process positive feedback information following PRB. By systematically weighing the expected maximum benefits influenced by various factors, they form a stable and unique pattern of PRB responses (Köster et al. 2022). Thus, pathway C facilitates the smooth execution and development of subsequent PRB. Impairment of this pathway may lead to a disruption in the individual established patterns of PRB responses, thereby resulting in the emergence and development of negative behavioral response patterns in prosocial risky dilemmas.

The dual‐system collaboration model of PRB, which is predicated on the dynamic interplay between emotional drive and cognitive reasoning, offers a fresh theoretical lens for understanding the mechanisms underlying PRB. This model not only uncovers the psychological and neural mechanisms by which individuals assess risks and benefits during PRB but also holds significant practical value for guiding moral education, crisis intervention, and social governance in real‐life contexts. Specifically, in the realm of moral education, the model allows for the precise identification of key factors influencing adolescent PRB, enabling the design of targeted educational interventions to cultivate proactive decision‐making skills in the face of prosocial risky dilemmas. For example, simulating prosocial risky dilemmas, enhances individual cognitive evaluations of loss aversion and guilt aversion, thereby increasing the likelihood of PRB in emergencies. In terms of crisis intervention, the model highlights the synergistic effects of emotional drive and cognitive reasoning, providing a theoretical foundation for crafting effective strategies to manage prosocial risk crises. Interventions such as emotional regulation training and cognitive restructuring can assist individuals in integrating emotional and cognitive resources more effectively during emergencies, leading to PRB that benefits others. At the societal governance level, the model facilitates the development of social mechanisms that encourage active citizen participation. By comprehending individual PRB preferences under specific conditions, incentives can be crafted to motivate public involvement in social welfare initiatives like public safety and environmental conservation, while ensuring personal safety, thus enhancing overall societal well‐being.

## 4 Research Paradigm of Prosocial Risky Behavior in the Economic Decision‐Making Domain and Cognitive Computational Modeling

### 4.1 Research Paradigm of Prosocial Risky Behavior in the Economic Decision‐Making Domain

As mentioned earlier, current research tools for PRB primarily consist of self‐report scales and dilemma‐priming paradigms within the social decision‐making domain (Skaar, Christ, and Jacobucci 2014; Dou et al. 2020; Liu et al. 2023). While these tools possess good reliability and validity and can be used under certain conditions to examine psychological characteristics, behaviors, and attitudes related to PRB, they also have drawbacks such as high subjectivity, lack of direct experience and genuine emotional investment, and limited applicability in the economic domain. Therefore, to address these shortcomings, it is necessary to design more refined PRB research paradigms from the perspective of economic decision‐making, which would better integrate with cognitive neuroscientific technologies (such as cognitive computational modeling), thereby facilitating a systematic and comprehensive examination of the underlying mechanisms of PRB.

Building on previous research (Cui et al. 2022; Gross et al. 2020), this paper has designed a risk‐assisting task with real monetary rewards. Specifically, in this research paradigm, each participant completes the experiment with a randomly recruited experimental assistant, with the participant acting as the decision‐maker and the assistant as the recipient. The decision‐maker is asked to decide whether to take the risk of financial loss to help another gain financial benefits. The task involves multiple rounds of binary decisions between an avoidance option (not helping, gaining *M* units of profit for oneself while the recipient gains nothing) and a risky help option (helping, but with a certain probability P1 of help failure; if failed, neither party gains any benefits, and if successful, both parties receive 90% of *M* units of profit). In this paradigm, helping is risky, with the risk probability P1 systematically varying between 0% and 100% across different trials, and the profit *M* of the avoidance option randomly varying between 10 units and 100 units in each trial. After deciding on each trial, participants receive feedback on the current decision outcome, successful or failed help, which matches the probability of help failure P1. The system provides monetary gains for both the decision‐maker and the recipient in the current trial on the decision result feedback interface. The final payment for the participant at the end of the experiment is the average of the monetary gains from all trials based on their decision feedback results.

### 4.2 Cognitive Computational Modeling of Prosocial Risky Behavior

Cognitive computing modeling technology has garnered widespread attention among researchers for its ability to quantify and reveal the underlying cognitive mechanisms of behavior. Specifically, this technology uses behavioral data to fit hypothetical models based on relevant statistical principles and conduct reliability analyses, thereby deriving the optimal formula that can accurately explain and predict individual internal cognitive processes. Based on the preliminary theoretical models and the dual‐system collaborative model of PRB (Bright and Goodman‐Delahunty 2006; Paxton and Greene 2010; Zhan et al. 2023), PRB involves the integrated processing of individual risk preferences and social preferences, reflecting the collaborative mechanism between emotional drive and cognitive reasoning. The loss/guilt aversion emotions induced by prosocial risky dilemmas, in conjunction with the cognitive appraisal of the subjective value of different options, jointly influence individual PRB reaction modes. The two types of aversion emotions are the subjective experiences of the individual two decision‐making preferences, and their intensity respectively reflects the level of risk preference (loss aversion) and social preference (guilt aversion) when engaging in PRB. Previous studies have utilized cognitive computing modeling technology to explore individual singular social preferences (such as guilt aversion) or risk preferences (such as loss aversion), transforming individual singular prosocial or risky behavioral responses (such as choices to help, adventurous choices) into internal decision preferences and the subjective value of decisions (Chen, Julien, and Dreher 2022; Gao and Zhou 2021; Moreira et al. 2021). Loss aversion refers to the phenomenon where an equal amount of loss has a greater impact than the benefit in decision‐making under risk, and it is a primary indicator of how individuals process risk information (Moreira et al. 2021). Guilt aversion refers to the phenomenon where the consequences of not helping have a greater impact than the costs of assisting, and it is an important indicator of how individuals process information involving helping situations (Carlson et al. 2022). For example, studies have examined individual levels of risk preference, finding that the higher the internal level of loss aversion, the more inclined people are to engage in risk‐avoidant behavior (Gross et al. 2020; Yu et al. 2022). Risk decision‐making is the outcome of a subjective trade‐off between unknown losses and the perceived value of taking risks (Gross et al. 2020). Moreover, previous research has quantified the level of internal guilt aversion in moral dilemmas and has found that individuals in emergencies exhibit higher levels of internal guilt aversion (Chen, Julien, and Dreher 2022). Therefore, this paper, integrating previous relevant research and theoretical models of PRB (Baar, Chang, and Sanfey 2019; Huebner, Dwyer, and Hauser 2009; Paxton and Greene 2010; Zhan et al. 2023), and in conjunction with the risk‐assisting task designed above, constructs the following computational models based on different theoretical assumptions:

(1) $$∆\mathrm{SV}=\left(1-\alpha \right)*\mathrm{EM}-\alpha *M$$

(2) $$∆\mathrm{SV}=\beta *\mathrm{EM}-\left(1-\beta \right)*M$$

(3) ∆SV=β*EM−α*M

(4) $$\mathrm{EM}=M*0.9*\left(1-P1\right)*2$$

(5) $$P2=\left(\frac{1}{1+e^{-\gamma ∆\mathrm{SV}}}\right)*\left(1-2\varepsilon \right)+\varepsilon$$

(6) $$\mathrm{AIC}=n\cdot \ln\;\left(\mathrm{SSE}/n\right)+k*2$$

Model 1 (loss aversion model) assumes that individuals adjust their behavioral responses based on the loss risk information in prosocial risky dilemmas, ignoring the anticipated value of PRB, the appeal for help from others, and their prosocial motivations. Model 2 (guilt aversion model) assumes that individuals adjust their behavioral responses based on the appeal for help from others and their prosocial motivations within prosocial risky dilemmas, neglecting the anticipated value of decisions and the unknown losses caused by risks in the dilemma. Model 3 (integrated processing model), building upon models 1 and 2, takes into account the synergistic mechanisms of cognitive reasoning and emotional drive in PRB. It hypothesizes that individuals, based on the principle of maximizing the subjective value derived from decisions, engage in cognitive reasoning regarding the negative emotional experiences evoked by dilemmas under different conditions and the cognitive appraisal of the subjective value of different options, thereby exhibiting different PRB reaction modes.

Specifically, SV represents the subjective value of a decision, which stems from an individual cognitive appraisal of the expected value of different options in prosocial risky dilemmas, weighted by the loss aversion parameter *α* and the guilt aversion parameter *β*. The decision criterion for individuals is to maximize SV. ΔSV is the difference in subjective value between the risky help option and the risk‐avoidant non‐help option; the larger this value, the higher the subjective value that participants assign to the risky help option, leading to a greater inclination to engage in PRB. Conversely, it indicates a preference for the risk‐avoidant non‐help option. The loss aversion parameter *α* represents the difference in subjective expected self‐gain and self‐loss between the risky help option and the risk‐avoidant non‐help option, with a higher value indicating a stronger tendency to avoid risk. The guilt aversion parameter *β* represents the difference in subjective expected collective gain and self‐loss between the risky help option and the risk‐avoidant non‐help option, with a higher value indicating a stronger prosocial inclination. P2 is the probability of participants choosing the risky help option in multiple rounds of decision‐making tasks. EM is the expected benefit upon successful help, influenced by the probability P1 of help failure, and *M* is the monetary amount received by participants for choosing the risk‐avoidant non‐help option in each round of decision‐making (Formula 4).

Moreover, this paper intends to employ the SoftMax function (Formula 5) to convert the differences in subjective decision values of participants across multiple rounds of tasks into the probability of choosing the risky help option (Baar, Chang, and Sanfey 2019). Here, *γ* represents the participant‐specific inverse temperature parameter, reflecting the sensitivity to decision values. The higher its value, the greater the certainty of the decision, which is associated with high confidence. The larger the temperature parameter, the smaller the difference in probabilities across categories output by the SoftMax function, and vice versa. *ε* reflects the choice noise due to irrelevant variables (such as distraction) that affect decision values. Non‐linear optimization is used to optimize participant‐specific parameters across all trials to obtain maximum likelihood estimates. Building on previous research, to better simulate and predict participants' decision‐making preferences and actual behaviors, computing models adjust the values of the loss aversion parameter *α* and the guilt aversion parameter *β* (0 < *α*, *β*) (Baar, Chang, and Sanfey 2019). They iteratively fit models' behavioral predictions against actual behaviors across all trials to identify the paired parameter values that minimize the sum of squared errors (minimizing the unexplained variance). To avoid terminating the fitting process at a local minimum, the computing models' fitting algorithm initializes at 10,000 random points within the *α*–*β* parameter space for each participant. In cases where computing model fitting results in a tie between two or more iterations during the fitting process, the best computing model fit that first appears is selected as the final computing result.

To compare the superiority of computing models of PRB proposed in this paper, following similar past studies (Baar, Chang, and Sanfey 2019), this paper intends to use the Akaike information criterion (AIC) criterion (Formula 6) to test its performance. This criterion can effectively verify the goodness of fit of cognitive computing models, determining whether the hypothesized cognitive computing model fits the actual observational data better and approximates reality more closely compared to other models. This paper did not choose the Bayesian information criterion (BIC) because the BIC assumes that the true generative computing model is within the set of candidates' computing models, measuring the degree to which one believes a certain computing model is the true data‐generating model, whereas the AIC does not assume that any candidate computing model is necessarily true. Thus, the AIC may be more suitable for this cognitive computing modeling concept. Moreover, this paper assumes that the errors of the three types of cognitive computing models follow a normal distribution, SSE represents the sum of squared residuals (the sum of the squares of the differences between the computing model's predictions and actual behavior), *n* represents the number of observations (trials), and *K* represents the number of free parameters in the computing model (*α*, *β*), quantifying the AIC values of the three types of computing models for each participant, and performing a paired rank test on the AIC values between two computing models to verify the optimality of the computing model.

**FIGURE 2:**
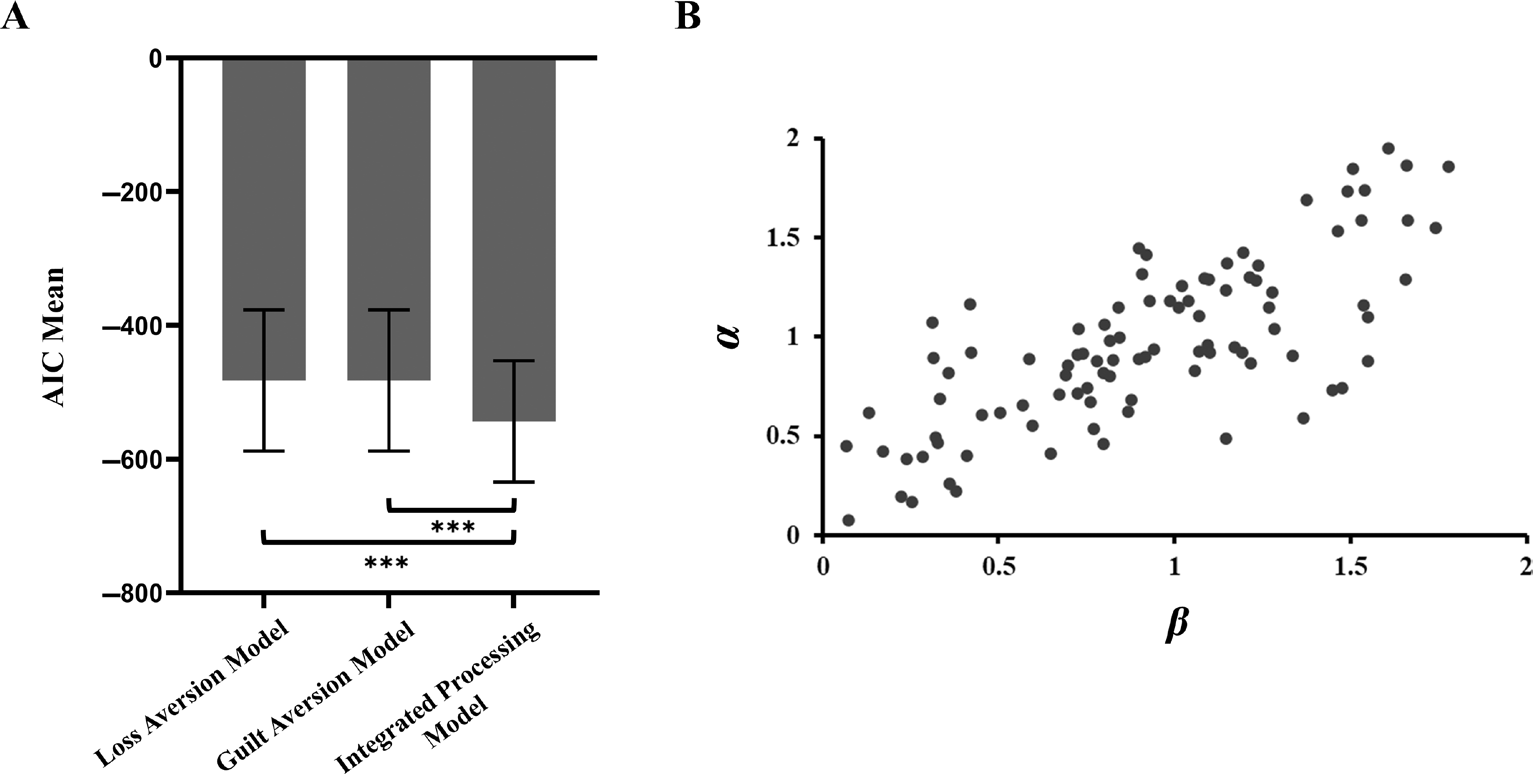
(A) Computing model comparison results. (B) Two‐dimensional distribution space of decision preference parameters. ***p < 0.001.

In addition, to preliminarily verify the feasibility of the cognitive computing modeling, recruited 100 participants to complete the risk‐assisting task, fitted their actual behavioral responses to three cognitive computational models, and compared the fitting results of the three models. The results indicated that the integrated processing computing model was the best fit, *R* ^2^ = 0.943. Compared to the integrated processing computing model, the AIC values of the guilt aversion computing model were significantly larger, ΔAIC = 52.84, *Z* = −8.58, *p* < 0.001, as were those of the loss aversion computing model, ΔAIC = 52.84, *Z* = −8.58, *p* < 0.001 (as shown in Figure 2A). Moreover, the two‐dimensional distribution space of the decision preference parameters for all participants indicates (as shown in Figure 2B), that different participants exhibit varying psychological characteristics in the risk‐assisting task, weighing the loss aversion and guilt aversion with different emphases.

## 5 Summary and Future Research Directions

### 5.1 Summary

In summary, clarifying the mechanisms underlying PRB helps to restrain and regulate negative human behavioral tendencies, further promoting a positive transformation. This paper first reviews and organizes the definitions of PRB, existing research tools, relevant theoretical models, and neural mechanisms, identifying the shortcomings in the field: namely, the insufficient explanatory power of current theoretical models, the limited cross‐domain applicability and low reliability of existing research paradigms, and a lack of research approaches that quantify the cognitive mechanisms of PRB. Second, based on the existing theoretical models of PRB and its neural mechanisms, this paper constructs a dual‐system collaborative model of PRB from the perspective of the interactive effects of emotional drive and cognitive reasoning. Third, based on the dual‐system collaborative model of PRB and research paradigms in the economic decision‐making domain (risk‐assisting task), this paper proposes a cognitive computational modeling approach for PRB and preliminarily verifies its reliability. Finally, from a cross‐cultural comparative perspective, this paper proposes research concepts regarding cultural differences in PRB from three aspects: cultural values, self‐concept, and cognitive orientation, thereby enhancing the cross‐cultural interpretive power of the constructed dual‐system collaborative model of PRB. This broadens the theoretical explanations for the mechanisms underlying PRB, expands the methodological dimensions for the study of PRB, and provides methodological and instrumental support for further revealing the cognitive mechanisms of PRB. On a practical level, it lays a theoretical foundation for the intervention of PRB.

### 5.2 Future Research Directions: Cross‐Cultural Differences in Prosocial Risky Behavior

Human emotional processing, cognitive reasoning, and behavioral responses are culturally sensitive and exhibit significant cultural differences (Hu, Yu, and Peng 2018). Regarding PRB, current research has largely focused on exploring individual differences and social effects (Dou et al. 2018; Song et al. 2018), with few studies discussing cultural differences in PRB and its underlying mechanisms. Therefore, the dual‐system collaborative model of PRB proposed in this paper requires further validation regarding cross‐cultural interpretive power. Future research should conduct cross‐cultural studies to explore the cultural differences in the collaborative mechanisms of emotional drive and cognitive reasoning for PRB within the comparative framework of Chinese and Western cultures, to test and enhance the cross‐cultural interpretive power of the dual‐system collaborative model of PRB constructed in this paper.

#### 5.2.1 Cultural Values

In cultural psychology research, theories are primarily distinguished by three orientations, values, self‐concept, and cognition. Among these, the value orientation is exemplified by the dichotomy of collectivism and individualism. A substantial body of research has indicated that the West tends to lean towards individualistic values, while the East is more inclined towards collectivist values (Huang, Sun, and Luo 2022). As a cultural system based on norms and values, individualism places greater emphasis on individual interests and goals, whereas collectivism places greater emphasis on the interests of the group and the coordination of those interests (Wei et al. 2023). Research has found that individualistic and collectivist values significantly influence individual single‐risk preferences or social preferences during decision‐making (Wang et al. 2017). For example, individuals who adhere to collectivist values pay greater attention to risk events, placing a higher value on aligning their interests, those of others, and the interests of the group (Kahan et al. 2017; Zhang, Ma, and Nater 2019). Individualism emphasizes self‐interest, leading to more interpersonal competition and conflict among such individuals. Moreover, when cooperating with in‐group members, higher activation in the left TPJ is not detected (Huang, Sun, and Luo 2022; Song et al. 2018), whereas individuals with collectivist values show the opposite pattern (Apanovich et al. 2017; Scarpati and Pina 2017). However, current research has paid less attention to the impact of collectivist/individualist values on PRB, especially their underlying mechanisms. Based on previous studies, this paper hypothesizes that compared to individuals with individualistic values, those with collectivist values may experience intense guilt aversion emotions when faced with the plight of others, thereby having a lower level of risk perception and being more inclined to engage in PRB.

#### 5.2.2 Self‐Concept

The representative of the self‐concept orientation is the distinction between the interdependent self and the independent self. Previous research has found that Easterners tend towards an interdependent self‐concept, while Westerners tend towards an independent self‐concept (Lehman, Chiu, and Schaller 2004). The interdependent self is a specific cultural pattern that emphasizes interpersonal relationships and internalizes the self‐concept as a significant member of the in‐group. In contrast, the independent self emphasizes the distinction of the self from others with clear interpersonal boundaries (Morris, Chiu, and Liu 2015). Individuals with different self‐concept orientations exhibit significant differences in decision‐making and psychological processes in risk or prosocial contexts. Research has found that individuals with an independent self‐concept place greater emphasis on their interests and inherent power than those with an interdependent self‐concept. Faced with unknown personal losses, such individuals are more inclined to avoid risk (Xue et al. 2014). However, compared to individuals with an independent self‐concept, those with an interdependent self‐concept pay more attention to their moral obligations and are inclined to sacrifice personal interests for the collective (Boer and Fischer 2013). Moreover, the results of a brain imaging study have confirmed that in terms of empathy and attribution, Chinese participants representing an interdependent self‐concept exhibited greater activation in the left parietal region and the left TPJ compared to American participants representing an independent self‐concept (Cheon et al. 2013). However, there is currently a lack of research directly examining the differences in PRB responses among individuals with different self‐orientations, making it difficult to reveal the cultural differences in their mechanisms. Previous research has found that participants with an interdependent self‐concept place greater emphasis on interpersonal relationships, such as those with family and friends (Ji 2008). Therefore, this paper hypothesizes that such individuals may exhibit a form of “interpersonal hierarchy,” where the closer the social distance to the person in need, the more intense the guilt aversion and the weaker the loss aversion emotions experienced by the individual. Subsequently, through cognitive trade‐offs, they are more inclined to engage in PRB. This can be explained by kin selection theory (Zhan et al. 2022), where such individuals engage in PRB towards relatives or friends to maintain kinship ties, which has evolutionary significance.

#### 5.2.3 Cognitive Orientation

In addition, cognitive orientation is characterized by the distinction between holistic and analytic thinking. This perspective holds that Easterners tend to adopt a holistic approach in cognitive processing, while Westerners favor an analytic approach. Holists believe that all things in the world are interconnected and interdependent, embracing an inclusive attitude towards all contradictions (Maddux and Yuki 2006; Masuda and Nisbett 2001), whereas analysts focus solely on the local attributes of things, using formal logic to cognitively process the external world (Spencer‐Rodgers, Peng, and Wang 2010). Based on these cognitive differences, individuals with these two orientations may exhibit different mechanisms when engaging in PRB. Previous studies have indicated that holists have a stronger prosocial motivation, emphasizing the wholeness of things, with no absolute opposition or contradiction (De et al. 2006). However, some research has found that, compared to holists, analysts are more inclined to finely process unknown losses and potential gains in risk situations, thereby making rational decisions by weighing the pros and cons, and there are differences in the FNR waves of the relevant brain regions (De et al. 2006; Van der Linden 2015). Based on this, this paper hypothesizes that the emotional drive and cognitive reasoning dual‐system collaborative mechanisms involved when holists and analysts engage in PRB may be significantly different. However, no research has yet been able to directly confirm the above research hypothesis.

Therefore, subsequent studies should utilize a variety of cognitive neuroscientific technologies (such as cognitive computing modeling, electrophysiology, and brain imaging) to systematically explore and compare the differences in the emotional drive and cognitive reasoning dual‐system collaborative mechanisms of PRB in Chinese and Western cultural contexts. This would facilitate an understanding of the diversity of PRB from the perspective of cultural differences, test and enhance the cross‐cultural interpretive power of the dual‐system collaborative model of PRB, and thereby contribute to revealing the deeper nature of human PRB.

## Conflicts of Interest

The authors declare no conflicts of interest.

## Data Availability

The original contributions presented in the study are included in the article, further inquiries can be directed to the corresponding author.

## References

[pchj822-bib-0001] Ajzen, I. 1991. “The Theory of Planned Behavior.” Organizational Behavior and Human Decision Processes 50, no. 2: 179–211. 10.1016/0749-5978(91)90020-t.

[pchj822-bib-0002] Apanovich, V. V. , B. N. Bezdenezhnykh , M. Sams , I. P. Inen , and Y. Alexandrov . 2017. “Event‐Related Potentials During Individual, Cooperative, and Competitive Task Performance Differ in Subjects With Analytic vs. Holistic Thinking.” International Journal of Psychophysiology 123: 136–142. 10.1016/j.ijpsycho.2017.10.001. 28986326

[pchj822-bib-0003] Arfer, K. B. , M. T. Bixter , and C. C. Luhmann . 2015. “Reputational Concerns, Not Altruism, Motivate Restraint When Gambling With Other People's Money.” Frontiers in Psychology 6: 848. 10.3389/fpsyg.2015.00848. 26157402 PMC4477537

[pchj822-bib-0004] Baar, J. M. v. , L. J. Chang , and A. G. Sanfey . 2019. “The Computational and Neural Substrates of Moral Strategies in Social Decision‐Making.” Nature Communications 10: 1483. 10.1038/s41467-019-09161-6. PMC6445121 30940815

[pchj822-bib-0006] Bault, N. , M. Joffily , A. Rustichini , and G. Coricelli . 2011. “Medial Prefrontal Cortex and Striatum Mediate the Influence of Social Comparison on the Decision Process.” Proceedings of the National Academy of Sciences of the United States of America 108, no. 38: 16044–16049. 10.1073/pnas.1100892108. 21896760 PMC3179055

[pchj822-bib-0007] Bereczkei, T. , B. Birkas , and Z. Kerekes . 2010. “Altruism Towards Strangers in Need: Costly Signaling in an Industrial Society.” Evolution and Human Behavior 31, no. 2: 95–103. 10.1016/j.evolhumbehav.2009.07.004.

[pchj822-bib-0008] Bereczkei, T. , B. Birkas , and Z. Kerekes . 2015. “The Presence of Others, Prosocial Traits, Machiavellianism: A Personality × Situation Approach.” Social Psychology 41, no. 4: 238–245. 10.1027/1864-9335/a000032.

[pchj822-bib-0009] Bixter, M. T. , and C. C. Luhmann . 2014. “Shared Losses Reduce Sensitivity to Risk: A Laboratory Study of Moral Hazard.” Journal of Economic Psychology 42, no. C: 63–73. 10.1016/j.joep.2013.12.004.

[pchj822-bib-0010] Blakemore, S. J. , and K. L. Mills . 2014. “Is Adolescence a Sensitive Period for Sociocultural Processing?” Annual Review of Psychology 1, no. 65: 187–207. 10.1146/annurev-psych-010213-115202. 24016274

[pchj822-bib-0011] Boer, D. , and R. Fischer . 2013. “How and When Do Personal Values Guide Our Attitudes and Sociality? Explaining Cross‐Cultural Variability in Attitude‐Value Linkages.” Psychological Bulletin 139, no. 5: 1113–1147. 10.1037/a0031347. 23339521

[pchj822-bib-0012] Bright, D. A. , and J. Goodman‐Delahunty . 2006. “Gruesome Evidence and Emotion: Anger, Blame, and Jury Decision‐Making.” Law and Human Behavior 30, no. 2: 183–202. 10.1007/s10979-006-9027-y. 16786406

[pchj822-bib-0085] Bucciarelli, M. , S. Khemlani , and P. N. Johnson‐Laird . 2008. “The Psychology of Moral Reasoning.” Judgment and Decision Making 3, no. 2: 121–139. 10.1017/S1930297500001479.

[pchj822-bib-0013] Carlson, R. W. , Y. E. Bigman , K. Gray , M. J. Ferguson , and M. J. Crockett . 2022. “How Inferred Motives Shape Moral Judgements.” Nature Reviews Psychology 1, no. 8: 468–478. 10.1038/s44159-022-00071-x.

[pchj822-bib-0014] Chen, P. , J. Qiu , H. Li , and Q. Zhang . 2009. “Spatiotemporal Cortical Activation Underlying Dilemma Decision‐Making: An Event‐Related Potential Study.” Biological Psychology 82, no. 2: 111–115. 10.1016/j.biopsycho.2009.06.007. 19576947

[pchj822-bib-0015] Chen, Q. , B. Julien , and J. Dreher . 2022. “Neurocomputational Mechanisms Engaged in Moral Choices and Moral Learning.” Neuroscience and Biobehavioral Reviews 132: 50–60. 10.1016/j.neubiorev.2021.11.023. 34826508

[pchj822-bib-0016] Chen, Y. , M. Xu , and X. Wang . 2020. “The Cognitive Neural Network Model of Trust.” Advances in Psychological Science 28, no. 5: 800–809. 10.3724/sp.j.1042.2020.00800.

[pchj822-bib-0017] Cheon, B. K. , D. M. Im , T. Harada , et al. 2013. “Cultural Modulation of the Neural Correlates of Emotional Pain Perception: The Role of Other‐Focusedness.” Neuropsychologia 51, no. 7: 1177–1186. 10.1016/j.neuropsychologia.2013.03.018. 23566889

[pchj822-bib-0018] Crockett, M. J. , Z. Kurth‐Nelson , J. Z. Siegel , P. Dayan , and R. J. Dolan . 2014. “Harm to Others Outweighs Harm to Self in Moral Decision Making.” Proceedings of the National Academy of Sciences of the United States of America 111, no. 48: 17320–17325. 10.1073/pnas.1408988111. 25404350 PMC4260587

[pchj822-bib-0019] Cui, F. , J. Yang , R. Gu , and J. Liu . 2022. “Functional Connectivities of the Right Temporoparietal Junction and Moral Network Predict Social Framing Effect: Evidence From Resting‐State fMRI.” Acta Psychologica Sinica 53, no. 1: 55–66. 10.3724/sp.j.1041.2021.00055.

[pchj822-bib-0020] De, M. B. , D. Kumaran , B. Seymour , and R. J. Dolan . 2006. “Frames, Biases, and Rational Decision‐Making in the Human Brain.” Science 313, no. 5787: 684–687. 10.1126/science.1128356. 16888142 PMC2631940

[pchj822-bib-0021] DeTienne, K. B. , C. F. Ellertson , M.‐C. Ingerson , and W. R. Dudley . 2021. “Moral Development in Business Ethics: An Examination and Critique.” Journal of Business Ethics 170, no. 3: 429–448. 10.1007/s10551-019-04351-0.

[pchj822-bib-0022] Do, K. T. , J. F. G. Moreira , and E. H. Telzer . 2017. “But Is Helping You Worth the Risk? Defining Prosocial Risk Taking in Adolescence.” Developmental Cognitive Neuroscience 25, no. C: 260–271. 10.1016/j.dcn.2016.11.008. 28063823 PMC5461219

[pchj822-bib-0023] Dolcos, F. , A. D. Iordan , and S. Dolcos . 2011. “Neural Correlates of Emotion‐Cognition Interactions: A Review of Evidence From Brain Imaging Investigations.” Journal of Cognitive Psychology 23, no. 6: 669–694. 10.1080/20445911.2011.594433. 22059115 PMC3206704

[pchj822-bib-0024] Dou, K. , Y. Huang , J. Li , and Y. Nie . 2020. “Validation of the Chinese Version of Prosocial Risky Behavior Scale in Adolescents.” China Journal of Health Psychology 28, no. 10: 1538–1542. 10.13342/j.cnki.cjhp.2020.10.023.

[pchj822-bib-0025] Dou, K. , Y. Liu , Y. Wang , and Y. Nie . 2018. “Willingness to Cooperate: Emotion Enhancement Mechanism of Perceived Social Mindfulness on Cooperative Behavior.” Acta Psychologica Sinica 50, no. 1: 101–114. 10.3724/sp.j.1041.2018.00101.

[pchj822-bib-0026] Fehr, E. , and U. Fischbacher . 2003. “The Nature of Human Altruism.” Nature 425, no. 6960: 785–791. 10.1038/nature02043. 14574401

[pchj822-bib-0027] Feng, X. , K. Dou , and Y. Tang . 2024. “Positive Parenting and Prosocial Risky Behavior in Adolescents: Testing a Moderated Mediation Model.” Psychological Development and Education 40, no. 5: 658–666. 10.16187/j.cnki.issn1001-4918.2024.0506.

[pchj822-bib-0028] Gao, Q. , and Y. Zhou . 2021. “Psychological and Neural Mechanisms of Trust Formation: A Perspective From Computational Modeling Based on the Decision of Investor in the Trust Game.” Advances in Psychological Science 29, no. 1: 178–189. 10.3724/sp.j.1042.2021.00178.

[pchj822-bib-0029] Gross, J. , N. S. Faber , A. Kappes , A. M. Nussberger , and C. D. Dreu . 2020. “When Helping Is Risky: Behavioral and Neurobiological Mechanisms of Prosocial Decisions Entailing Risk.” Psychological Science 32, no. 11: 1842–1855. 10.31234/osf.io/n4wqd. PMC7614101 34705578

[pchj822-bib-0030] Gu, R. , J. Liu , and F. Cui . 2019. “Pain and Social Decision‐Making: New Insights From the Social Framing Effect.” Brain Science Advances 5, no. 4: 221–238. 10.26599/bsa.2019.9050020.

[pchj822-bib-0031] Haidt, J. 2001. “The Emotional Dog and Its Rational Tail: A Social Intuitionist Approach to Moral Judgment.” Psychological Review 108, no. 4: 814–834. 10.1037/0033-295x.108.4.814. 11699120

[pchj822-bib-0032] Hare, T. A. , N. Tottenham , A. Galvan , H. U. Voss , G. H. Glover , and B. J. Casey . 2008. “Biological Substrates of Emotional Reactivity and Regulation in Adolescence During an Emotional Go‐Nogo Task.” Biological Psychiatry: Cognitive Neuroscience and Neuroimaging 63, no. 10: 927–934. 10.1016/j.biopsych.2008.03.015. PMC2664095 18452757

[pchj822-bib-0033] Hu, X. , F. Yu , and K. Peng . 2018. “How Does Culture Affect Morality? The Perspectives of Between‐Culture Variations, Within‐Culture Variations, and Multiculturalism.” Advances in Psychological Science 26, no. 11: 2081–2090. 10.3724/sp.j.1042.2018.02081.

[pchj822-bib-0034] Huang, L. , Y. Sun , and S. Luo . 2022. “The Impact of Individualism on the Efficiency of Epidemic Control and the Underlying Computational and Psychological Mechanisms.” Acta Psychologica Sinica 54, no. 5: 497–515. 10.3724/sp.j.1041.2022.00497.

[pchj822-bib-0035] Huebner, B. , S. Dwyer , and M. Hauser . 2009. “The Role of Emotion in Moral Psychology.” Trends in Cognitive Sciences 13, no. 1: 1–6. 10.1016/j.tics.2008.09.006. 19058993

[pchj822-bib-0036] Hursh, S. R. , and P. G. Roma . 2013. “Behavioral Economics and Empirical Public Policy.” Journal of the Experimental Analysis of Behavior 42, no. 3: 435–452. 10.1002/jeab.7. 23344991

[pchj822-bib-0037] Ji, L. J. 2008. “The Leopard Cannot Change His Spots, or Can He? Culture and the Development of Lay Theories of Change.” Personality and Social Psychology Bulletin 34, no. 5: 613–622. 10.1177/0146167207313935. 18322267

[pchj822-bib-0038] Kahan, D. M. , K. H. Jamieson , A. R. Landrum , and K. Winneg . 2017. “Culturally Antagonistic Memes and the Zika Virus: An Experimental Test.” Social Science Electronic Publishing 20: 1–4. 10.1080/13669877.2016.1260631.

[pchj822-bib-0039] Köster, R. , D. Hadfield‐Menell , R. Everett , L. Weidinger , G. K. Hadfield , and J. Z. Leibo . 2022. “Spurious Normativity Enhances Learning of Compliance and Enforcement Behavior in Artificial Agents.” Proceedings of the National Academy of Sciences 119, no. 3: e2106028118. 10.1073/pnas.2106028118. PMC8784148 35022231

[pchj822-bib-0040] Lehman, D. R. , C. Y. Chiu , and M. Schaller . 2004. “Psychology and Culture.” Annual Review of Psychology 55, no. 1: 689–714. 10.1146/annurev.ps.24.020173.002035. 14744231

[pchj822-bib-0041] Liu, C. , X. Xiao , Q. Pi , Q. Tan , and Y. Zhan . 2023. “Are You More Risk‐Seeking When Helping Others? Effects of Situational Urgency and Peer Presence on Prosocial Risky Behavior.” Frontiers in Psychology 14: 1036624. 10.3389/fpsyg.2023.1036624. 36935944 PMC10020997

[pchj822-bib-0042] Locke, E. A. , and G. P. Latham . 2002. “Building a Practically Useful Theory of Goal Setting and Task Motivation.” American Psychologist 57, no. 9: 705–717. 10.1037/0003-066x.57.9.705. 12237980

[pchj822-bib-0043] Maddux, W. W. , and M. Yuki . 2006. “The Ripple Effect: Cultural Differences in Perceptions of the Consequences of Events.” Personality and Social Psychology Bulletin 32, no. 5: 669–683. 10.1177/0146167205283840. 16702159

[pchj822-bib-0044] Masuda, T. , and R. E. Nisbett . 2001. “Attending Holistically Versus Analytically: Comparing the Context Sensitivity of Japanese and Americans.” Journal of Personality and Social Psychology 81, no. 5: 922–934. 10.1037/0022-3514.81.5.922. 11708567

[pchj822-bib-0045] Minwoo, L. , S. Sunhae , and K. Hackjin . 2018. “Social Observation Increases Deontological Judgments in Moral Dilemmas.” Evolution and Human Behavior 39, no. 6: 611–621. 10.1016/j.evolhumbehav.2018.06.004.

[pchj822-bib-0046] Moreira, J. , S. M. Tashjian , A. Galván , and J. A. Silvers . 2021. “Computational and Motivational Mechanisms of Human Social Decision Making Involving Close Others.” Journal of Experimental Social Psychology 93, no. 5: 104086. 10.1016/j.jesp.2020.104086. 35291212 PMC8920070

[pchj822-bib-0047] Morris, M. W. , C.‐Y. Chiu , and Z. Liu . 2015. “Polycultural Psychology.” Annual Review of Psychology 66, no. 1: 631–659. 10.1146/annurev-psych-010814-015001. 25251481

[pchj822-bib-0048] Paramita, W. , F. Septianto , and F. Tjiptono . 2020. “The Distinct Effects of Gratitude and Pride on Donation Choice and Amount.” Journal of Retailing and Consumer Services 53: 101972. 10.1016/j.jretconser.2019.101972.

[pchj822-bib-0049] Paxton, J. M. , and J. D. Greene . 2010. “Moral Reasoning: Hints and Allegations.” Topics in Cognitive Science 2, no. 3: 511–527. 10.1111/j.1756-8765.2010.01096.x. 25163874

[pchj822-bib-0050] Qu, Y. , A. Galvan , A. J. Fuligni , M. D. Lieberman , and E. H. Telzer . 2015. “Longitudinal Changes in Prefrontal Cortex Activation Underlie Declines in Adolescent Risk Taking.” Journal of Neuroscience 35, no. 32: 11308–11314. 10.1523/jneurosci.1553-15.2015. 26269638 PMC4532760

[pchj822-bib-0051] Rand, D. G. , Z. G. Epstein , and B. Thomas . 2014. “Risking Your Life Without a Second Thought: Intuitive Decision‐Making and Extreme Altruism.” PLoS One 9, no. 10: e109687. 10.1371/journal.pone.0109687. 25333876 PMC4198114

[pchj822-bib-0052] Sarlo, M. , L. Lotto , A. Manfrinati , R. Rumiati , G. Gallicchio , and D. Palomba . 2012. “Temporal Dynamics of Cognitive‐Emotional Interplay in Moral Decision‐Making.” Journal of Cognitive Neuroscience 24, no. 4: 1018–1029. 10.1162/jocn_a_00146. 21981668

[pchj822-bib-0053] Scarpati, A. S. , and A. Pina . 2017. “Cultural and Moral Dimensions of Sexual Aggression: The Role of Moral Disengagement in Men's Likelihood to Sexually Aggress.” Aggression and Violent Behavior 37: 115–121. 10.1016/j.avb.2017.09.001.

[pchj822-bib-0054] Sevinc, G. , and R. N. Spreng . 2014. “Contextual and Perceptual Brain Processes Underlying Moral Cognition: A Quantitative Meta‐Analysis of Moral Reasoning and Moral Emotions.” PLoS One 9, no. 2: e87427. 10.1371/journal.pone.0087427. 24503959 PMC3913597

[pchj822-bib-0055] Skaar, N. R. , T. J. Christ , and R. Jacobucci . 2014. “Measuring Adolescent Prosocial and Health Risk Behavior in Schools: Initial Development of a Screening Measure.” School Mental Health: A Multidisciplinary Research and Practice Journal 6, no. 2: 137–149. 10.1007/s12310-014-9123-y.

[pchj822-bib-0056] Song, Y. , F. Ding , W. Shi , and X. Chen . 2018. “Perspective of Cultural Differences on the Formation Mechanism Construction and the Neural Basis of Individual Cooperative Behavior.” Journal of Psychological Science 41, no. 5: 1227–1232.

[pchj822-bib-0057] Song, Y. , R. Xu , and C. Xing . 2017. “Risk Sensitivity Theory: Need to Drive Risk Decisions.” Advances in Psychological Science 25, no. 3: 486–499. 10.3724/sp.j.1042.2017.00486.

[pchj822-bib-0058] Spencer‐Rodgers, J. , K. Peng , and L. Wang . 2010. “Dialecticism and the Co‐Occurrence of Positive and Negative Emotions Across Cultures.” Journal of Cross‐Cultural Psychology 50, no. 1: 293–298. 10.1007/bf02490831.

[pchj822-bib-0059] Tan, C. , P. Wang , and Y. Cui . 2017. “Should I Sacrifice My Profit Before His Eyes? Partner's Ability and Social Distance Affecting the Tendency of Reputation‐Profit Game.” Acta Psychologica Sinica 49, no. 9: 1206–1218. 10.3724/sp.j.1041.2017.01206.

[pchj822-bib-0060] Telzer, E. H. 2016. “Dopaminergic Reward Sensitivity Can Promote Adolescent Health: A New Perspective on the Mechanism of Ventral Striatum Activation.” Developmental Cognitive Neuroscience 17, no. C: 57–67. 10.1016/j.dcn.2015.10.010. 26708774 PMC4727991

[pchj822-bib-0061] Van der Linden, S. 2015. “A Conceptual Critique of the Cultural Cognition Thesis.” Science Communication 38: 128–138. 10.1177/1075547015614970.

[pchj822-bib-0062] Van Knippenberg, D. L. , R. Van Dick , and S. Tavares . 2010. “Social Identity and Social Exchange: Identification, Support, and Withdrawal From the Job.” Journal of Applied Social Psychology 37, no. 3: 457–477. 10.1111/j.1559-1816.2007.00168.x.

[pchj822-bib-0063] Vieira, J. B. , S. Schellhaas , E. Enstrm , and A. Olsson . 2020. “Help or Flight? Increased Threat Imminence Promotes Defensive Helping in Humans.” Proceedings of the Royal Society: Biological Sciences 287, no. 1933: 20201473. 10.31234/osf.io/bckn3. 32842931 PMC7482272

[pchj822-bib-0064] Volz, L. J. , B. L. Welborn , M. S. Gobel , M. S. Gazzaniga , and S. T. Grafton . 2017. “Harm to Self Outweighs Benefit to Others in Moral Decision Making.” Proceedings of the National Academy of Sciences of the United States of America 114, no. 30: 7963–7968. 10.1073/pnas.1706693114. 28696302 PMC5544327

[pchj822-bib-0065] Vugt, M. , and W. Iredale . 2013. “Men Behaving Nicely: Public Goods as Peacock Tails.” British Journal of Psychology 104, no. 1: 3–13. 10.1111/j.2044-8295.2011.02093.x. 23320439

[pchj822-bib-0066] Wang, X. , H. Zhang , D. Wu , and X. Lv . 2017. “Cultural Influences on Individual Risk Perception: Cultural Cognition Theory's Explanation.” Advances in Psychological Science 25, no. 8: 1251–1260. 10.3724/sp.j.1042.2017.01251.

[pchj822-bib-0067] Wei, X. , K. Zhang , X. Fu , and F. Wang . 2023. “Honor Culture and Face Culture: A Comparison Through the Lens of the Dignity, Honor, and Face Cultural Framework and Indigenous Social Theory.” Advances in Psychological Science 31, no. 8: 1541–1552. 10.3724/SP.J.1042.2023.01541.

[pchj822-bib-0068] Xie, X. , and J. Lu . 2014. “Double Reference Points in Risky Decision Making.” Advances in Psychological Science 22, no. 4: 571–579. 10.3724/sp.j.1042.2014.00571.

[pchj822-bib-0070] Xue, W. , D. W. Hine , N. M. Loi , E. B. Thorsteinsson , and W. J. Phillips . 2014. “Cultural Worldviews and Environmental Risk Perceptions: A Meta‐Analysis.” Journal of Environmental Psychology 40: 249–258. 10.1016/j.jenvp.2014.07.002.

[pchj822-bib-0071] Yu, H. , L. S. Contreras‐Huerta , A. M. B. Prosser , et al. 2022. “Neural and Cognitive Signatures of Guilt Predict Hypocritical Blame.” Psychological Science 33, no. 11: 1909–1927. 10.1177/09567976221122765. 36201792

[pchj822-bib-0072] Yudkin, D. A. , A. M. B. Prosser , S. M. Heller , K. McRae , A. Chakroff , and M. J. Crockett . 2022. “Prosocial Correlates of Transformative Experiences at Secular Multi‐Day Mass Gatherings.” Nature Communications 13: 2600. 10.1038/s41467-022-29600-1. PMC9142525 35624086

[pchj822-bib-0073] Zanon, M. , G. Novembre , N. Zangrando , L. Chittaro , and G. Silani . 2014. “Brain Activity and Prosocial Behavior in a Simulated Life‐Threatening Situation.” NeuroImage 98: 134–146. 10.1016/j.neuroimage.2014.04.053. 24780697

[pchj822-bib-0074] Zhan, Y. , C. Liu , X. Xiao , Q. Tan , and X. Fu . 2023. “Theoretical Models and Neural Mechanisms of Prosocial Risky Behavior.” Chinese Science Bulletin 68, no. Z1: 154–168. 10.1360/tb-2022-0699.

[pchj822-bib-0075] Zhan, Y. , X. Xiao , J. Li , et al. 2018. “Interpersonal Relationship Modulates the Behavioral and Neural Responses During Moral Decision‐Making.” Neuroscience Letters 672, no. 13: 15–21. 10.1016/j.neulet.2018.02.039. 29471002

[pchj822-bib-0076] Zhan, Y. , X. Xiao , Q. Tan , J. Li , and Y. Zhong . 2020. “Neural Correlations of the Influence of Self‐Relevance on Moral Decision‐Making Involving a Trade‐Off Between Harm and Reward.” Psychophysiology 9, no. 4: e13590. 10.1111/psyp.13590. 32324300

[pchj822-bib-0077] Zhan, Y. , X. Xiao , Q. Tan , J. Li , and Y. Zhong . 2022. “Influence of Reputational Concern and Social Distance on Moral Decision‐Making Under the Harmful Dilemma: Evidence From Behavioral and ERPs Study.” Acta Psychologica Sinica 54, no. 6: 613–627.

[pchj822-bib-0078] Zhan, Y. , X. Xiao , Q. Tan , and Y. Zhong . 2020. “Influence of Self‐Relevance on Moral Decision‐Making Under Reputational Loss Risk: An ERP Study.” Chinese Science Bulletin 65, no. 19: 1996–2009. 10.1360/tb-2019-0618.

[pchj822-bib-0079] Zhang, H. , H. Zhu , X. Jia , and C. Ma . 2024. “The Influence of Subjective Social Status on Adolescents' Prosocial Risk‐Taking Behavior: A Regulated Chain Mediation Model.” Psychological Development and Education 40, no. 4: 488–498. 10.16187/j.cnki.issn1001-4918.2024.04.04.

[pchj822-bib-0080] Zhang, Q. , J. Ma , and U. M. Nater . 2019. “How Cortisol Reactivity Influences Prosocial Decision‐Making: The Moderating Role of Sex and Empathic Concern.” Frontiers in Human Neuroscience 13: 415. 10.3389/fnhum.2019.00415. 32038194 PMC6988811

[pchj822-bib-0081] Zhang, Y. , Z. Yu , and X. Mai . 2020. “The Influence of Social Value Orientation on Self‐Other Risk Decision‐Making and Its Mechanisms.” Acta Psychologica Sinica 52, no. 7: 895–908. 10.3724/sp.j.1041.2020.00895.

[pchj822-bib-0082] Zheng, H. , R. Chen , and X. Mai . 2024. “The Cognitive and Neural Mechanism of Third‐Party Punishment.” Advances in Psychological Science 32, no. 2: 398–412. 10.3724/SP.J.1042.2024.00398.

[pchj822-bib-0083] Zhong, Y. , Y. Zhan , J. Li , and W. Fan . 2018. “Study on the Mechanism and Intervention of Moral Decision: Effects of Self‐Relevance and Risk Level.” Advances in Psychological Science 25, no. 7: 1093–1102. 10.3724/sp.j.1042.2017.01093.

[pchj822-bib-0084] Zinchenko, O. , V. Nikulin , and V. Klucharev . 2021. “Wired to Punish? Electroencephalographic Study of the Restingstate Neuronal Oscillations Underlying Third‐Party Punishment.” Neuroscience 471: 1–10. 10.1016/j.neuroscience.2021.07.012. 34302905

